# The Impact of Diabetes on Vascular Disease: Progress from the Perspective of Epidemics and Treatments

**DOI:** 10.1155/2022/1531289

**Published:** 2022-04-08

**Authors:** Runyang Liu, Lihua Li, Chen Shao, Honghua Cai, Zhongqun Wang

**Affiliations:** ^1^Department of Cardiology, Affiliated Hospital of Jiangsu University, Zhenjiang, China; ^2^Department of Pathology, Affiliated Hospital of Jiangsu University, Zhenjiang, China; ^3^Department of Burn Surgery, Affiliated Hospital of Jiangsu University, Zhenjiang, China

## Abstract

At present, the global incidence of diabetes has increased in countries with large populations, and the changes in developing regions are particularly worthy of attention. In the past 40 years or so, the income situation in China, India, and other countries has exploded, leading to changes in the way of life and work as well as an increase in the prevalence of diabetes. Metabolic disorders caused by diabetes can lead to secondary vascular complications, which have long-term malignant effects on the heart, kidneys, brain, and other vital organs of patients. Adequate primary prevention measures are needed to reduce the incidence of diabetic vascular complications, and more attention should be given to treatment after the disease. To this end, it is necessary to determine a standardized drug and physical therapy system and to build a more efficient and low-cost chronic disease management system.

## 1. Introduction

Diabetes can cause vascular complications directly or indirectly through a variety of mechanisms. Vascular lesions caused by diabetes include macrovascular and microvascular lesions. Macrovascular lesions may lead to cerebrovascular disease, cardiovascular disease, and peripheral vascular disease. Microvascular diseases can cause specific complications, such as renal failure, retinopathy, and neuropathy. In recent decades, the global incidence of diabetes has been on the rise, and coupled with the growth of the population base, the number of people with diabetes has gradually increased. Different from diabetes itself, the complications of diabetes are closely related to the region. In the past 30 years, the incidence of diabetes in traditional developed countries has been relatively high, but thanks to better diagnosis, treatment, and nursing processes, the incidence of diabetes complications has been low. However, in developing countries and regions, poor diagnosis and a general lack of nursing awareness has led to a high incidence of both diabetes and diabetes complications. Healthcare systems around the world carry an enormous financial burden to treat these complications. In this review, based on populations in different regions of the world, we analyzed the risk factors affecting diabetes and vascular complications and the prevalence of representative complications. We also summarized the prevention and treatment experience in many countries as well as reasonable prevention, treatment, and chronic disease management measures.

## 2. Epidemiology

According to estimates from the 9th edition of the IDF Diabetes Atlas in 2019, there are already approximately 463 million people with diabetes in the world, including estimated undiagnosed people, and based on risk factors, the prevalence of diabetes will continue to rise in the future. By 2030, an estimated 10.2% of the total population or 578 million people is expected to have diabetes. By 2045, this proportion will rise to 10.9%, and the total number of patients will reach 700 million, and increase of 51.9% compared to 2019 [1]. Similar incidence changes can also be found in smaller areas. A study evaluating the subsequent impact of diabetes in the United States noted that between 1990 and 2010, adults with diabetes increased 3.1-fold to 20.7 million people [2]. Similar to the United States, as of 2013, seven nationwide spot checks on the prevalence of diabetes in China showed that the number of patients diagnosed with diabetes in the past 40 years had exploded. On the one hand, this is due to the large population base in China. On the other hand, it has a strong relationship with the rising prevalence of diabetes [3–7].

The real scary thing about diabetes is not the disease itself but the complications it brings. Among adults with diabetes worldwide (20-79 years), the leading cause of death can be attributed to diabetic vascular complications [8]. Interestingly, there are large regional differences in the incidence of the vascular complications of diabetes. Researchers in the United States have pointed out that the prevalence of diabetic vascular complications, especially cardiovascular diseases, is experiencing a downward trend. Similar conclusions were also found in studies of cerebrovascular diseases, peripheral vascular diseases, and retinopathy [2, 9, 10]. Numerous European studies have shown similar conclusions, i.e., improvements in the prevalence of vascular complications as well as cardiovascular mortality [11, 12]. In some Asian countries, such as Japan and South Korea, whose economic situation is similar to that of Europe and the United States, the number of patients receiving drug treatment for diabetes is high, and nursing care for diabetes is also better than that in developing countries [13–15]. In relatively underdeveloped regions such as China, there is a relative lack of aggregated epidemiological reports on diabetic vascular complications, and most of them are regional sample surveys. Seven national surveys in China have also tended to focus on blood samples. Pooled surveys of vascular complications are typically cross-sectional, making it difficult to summarize trends in complications [16]. In India, surveys have also focused on the prevalence of diabetes, showing a growth trend similar to that in China. Surveys on vascular complications have also tended to focus on a specific time, and there is a lack of cohort studies and follow-up studies [17].

Therefore, it is difficult to draw direct and clear conclusions on the incidence of diabetic vascular complications in these developing countries.

## 3. The Influencing Factors of Diabetic Vascular Complications

### 3.1. External Factors

#### 3.1.1. Diet

The current popular modern diet features American fast food with good taste [18]. The diet of many developing countries has also undergone tremendous changes, with many Asian countries gradually shifting to a Western diet [19, 20]. This change in dietary trends will increase the incidence of obesity and, in turn, the incidence of type 2 diabetes globally. A prospective cohort study in the United States found that a Western dietary pattern was positively associated with cardiovascular disease, with the highest risk group having a nearly 2-fold increase in the incidence of CHD [21].

The reason modern diets have such an effect on the body is that they cause individuals to consume more carbohydrates, which plays a crucial role in the pathogenesis of diabetes. The original staple food in many parts of China is mainly coarsely processed grains other than rice. However, currently, Chinese consumers often refuse to eat coarse grains because of their higher price and poor taste. Refined grains have become the mainstream. This staple food has a higher glycemic index and less fiber, potentially increasing the risk of type 2 diabetes [22]. The role of added sugars, especially fructose, in beverages in the pathogenesis of diabetes has been increasingly emphasized [23]. A national survey conducted in the United States showed a linear correlation between fructose intake and the incidence of diabetes. The main reason for this change can be attributed to sugar-sweetened beverages, which account for an astonishing 50% of added sugar in the United States, a trend that has also spread to developing countries such as China and India [22, 24–26].

Western diets also consist of higher fat and protein intake, with protein intake tending to be dominated by red and processed meat [19]. Some Asian countries that are in transition also show a higher intake of fat, while the proportion of high-quality protein intake is relatively low [24]. Although there is little research on the subject, taking China as an example, the ratio of fat to protein in local food and beverages has also changed, and there is also a trend toward high-fat and highly processed meat. Among them, the contents of sodium glutamate and sodium chloride are high, such as in hot pot and Sichuan dishes. All of these factors increase the risk of diabetes and cardiovascular disease.

In addition, the effect of alcohol intake on macrovascular and microvascular complications has also been widely discussed. A Chinese study on hospitalized patients with type 2 diabetes noted a clear dose–response relationship between alcohol consumption and lower extremity aortic lesions, and the group that had drank alcohol for more than 20 years was 3.5 times more likely to develop the disease [27]. However, a case study in Singapore also showed that alcohol intake can reduce the incidence of diabetic retinopathy, suggesting that certain amounts of alcohol could have a protective effect on microvascular function [28].

#### 3.1.2. Physical Activity

Due to changes in production methods in developing countries, the proportion of manual labor in social production has declined. The continuous increase in motor vehicle usage has reduced the use commuting methods that require exercise, such as walking and cycling, as has relatively extended working hours and increases in sedentary time. Generally, people, especially urban residents, have less time for exercise [29].

In addition, sedentary activities such as watching TV account for a large proportion of the current entertainment patterns of urban residents in various countries. Such changes in work and play increase the risk of type 2 diabetes and even have a certain correlation with the genetics of obesity [30, 31].

#### 3.1.3. Other Lifestyle Factors

Smoking is an important risk factor for diabetes. At the beginning of the 21st century, a sample survey in China showed that up to 70% of men smoked. The prevalence of diabetes in smokers is approximately 20% higher compared with nonsmokers, with higher risk for those who started smoking earlier and those who have smoked longer. Older people are more likely to be at risk of developing diabetes [32]. At the same time, increases in environmental pollutants such as air pollutants and some harmful metal elements may also be correlated with increases in cerebrovascular complications, but whether air pollutants are related to type 2 diabetes remains to be confirmed [33].

### 3.2. Internal Factors

#### 3.2.1. Blood Sugar

In diabetic patients with poor glycemic control, some arteries in the body are more likely to have increased calcification and medial thickness and dilation, which ultimately leads to complete disease of the enlarged vessels. Worsening macrovascular disease is positively correlated with blood glucose levels, and the glucotoxicity and lipotoxicity caused by hyperglycemia and metabolic disorder, respectively, can lead to accelerated arteriosclerosis [18]. In T1DM patients, HbA1c (glycosylated glycemic protein) plays a crucial role in the assessment of blood glucose and is a leading risk factor for CVD in addition to age [27]. Rawshani's research also pointed out that the HbA1c level of diabetic patients is the strongest risk factor for stroke among many other factors [34].

However, lower blood sugar is not always better, and the effect of hypoglycemia on blood vessels has also been mentioned in many studies. From a mechanistic point of view, a small-scale blood glucose test study of 121 T2DM patients conducted by the University of Malta found a correlation between hypoglycemia and macrovascular disease stress or a series of inflammatory cytokine responses [35]. From an epidemiological point of view, a study that aggregated data from 109 countries showed that some countries in the Americas had the highest proportion of hypoglycemia-related mortality [12]. This suggests that large fluctuations in blood sugar and the impact of hypoglycemia on vascular complications among diabetic patients should not be ignored.

#### 3.2.2. Blood Lipids

It is well known that vascular fat deposition is a basic feature of macrovascular disease, and this also applies to macrovascular disease secondary to diabetes. Experiments based on LDL receptor-deficient mice have shown that blood lipids can actually mediate vascular lesions; the mechanism may be that lipids can induce downstream vascular-related signaling pathways [36]. The main components of atherosclerotic plaques are free cholesterol and cholesterol esters. The DCCT/EDIC study in Pittsburgh, USA, also found that vascular complications were associated with triglyceride (TG) levels. If TG/high-density lipoprotein cholesterol (HDL − C) > 2, the patient's cardiovascular risk is increased [37]. In addition to blood lipids, perivascular adipose tissue (PAT) can cause chemotaxis among inflammatory cells through a variety of inflammatory factors and can destroy the normal tension of blood vessels through the insulin signaling pathway, thereby impairing vascular function, aggravating insulin resistance, and increasing the occurrence of vascular complications [38].

#### 3.2.3. Blood Pressure

Both Eastern and Western scholars have found a positive correlation between hypertension and diabetic vascular complications. A prospective cohort study in Finland found that from 1995 to 2008, patients with type 1 diabetes were more likely to suffer from coronary heart disease and stroke and have a higher mortality rate due to complications [39]. The China Stroke Primary Prevention Trial (CSPPT) conducted in China found that during the 4.5-year survey period, subjects with a systolic blood pressure between 130 and 140 mmHg had a 1.37-fold higher risk of developing diabetes than the 120-130 mmHg group, and hypertension reduced the probability of the former group returning to a normal fasting blood sugar by approximately 30% [40].

#### 3.2.4. BMI/Waist Circumference

A high BMI is often accompanied by a higher risk of diabetes. A prospective study conducted by Wang showed that a higher BMI was the most important factor related to the onset of diabetes. The prevalence of diabetes among Chinese individuals with a higher BMI can reach nearly twice than that among individuals with a normal BMI in the same period [41]. Cohort studies conducted in the United States from 2007 to 2012 pointed out that the onset of diabetes may not be related to body mass index alone and that a larger waist circumference may be more closely related to the onset of diabetes [42]. This point may be more applicable in Asian populations. A study conducted in southern China found that the obesity rate among 15,364 respondents was 7.9% but the rate of abdominal obesity was up to 1.3% among people with a low BMI. This is because, for many individuals in China, obesity typically manifests as central obesity characterized by fat accumulation in the abdomen, and because BMI cannot indicate local fat distribution, it is often lower in Asian diabetic patients [43].

However, a BMI that is too low does not play a protective role against the onset of diabetic complications. A study conducted by Zhang in China with 3,224 patients showed that although diabetic retinopathy (DR) and atherosclerotic plaques were positively correlated with BMI, the incidence of diabetic neuropathy presented a U-shaped curve with increased BMI, indicating that a BMI that is too low or too high may increase the possibility of diabetes onset [23].

### 3.3. New Challenges

#### 3.3.1. Regional and Ethnic Differences

Many countries are relatively ethnically diverse, and interindividual differences in diabetes prevalence may be partly attributable to ethnic differences. The U.S. has many immigrants, so studies can compare genetic differences in diabetes prevalence in the same environment. A study of 49,574 subjects showed that between 1997 and 2004, racial differences in diabetes prevalence were found among different BMI groups, with the largest racial differences among the normal BMI groups, and this gap shows a widening trend [44]. The prevalence of diabetes among different ethnic groups in China has also been mentioned by many scholars. For example, the prevalence of diabetes in Tibetans is significantly lower than that in Han Chinese people. Apart from the differences in economic and lifestyle factors analyzed previously, Tibetans are more likely to have diabetes. High exercise levels may prevent the vascular complications of diabetes [3, 45]. Racial differences can also be reflected in specific complications. A study comparing different ethnic groups in Singapore showed that Singaporeans of Indian descent had a higher risk of developing diabetic eye disease than those of Chinese and Malayan descent (Indian 30.7%, Chinese 26.2%, Malayan 25.5%, *p* = 0.012) [46]. Gupta and Misra's study also found that South Asians had a higher incidence of retinopathy than Caucasians [47].

In addition to differences between races and countries, differences in prevalence within the same country are also worthy of attention. A Chinese study that aggregated data from 31 items showed that the prevalence of diabetes was 1.73 times higher in eastern regions compared with western regions [48]. An urban-rural disparity in diabetes in Asian countries is also increasingly evident. Urban areas have a higher incidence and better medical standards. In contrast, the incidence of diabetes in rural areas is lower, but medical care is less optimum, resulting in a greater incidence of vascular complications. Postmortem mortality is also higher [17, 49, 50].

#### 3.3.2. Juvenile Onset

Diabetes, especially type 2 diabetes, shows a trend of younger onset, and it is more characteristic for adolescents to have an increased incidence of diabetes [51]. Because young diabetic patients suffer from early disease onset, the course of diabetes is greatly prolonged, and the possibility of cardiovascular complications is greatly increased. This trend creates a huge burden in terms of the diagnosis and treatment of vascular complications [52].

#### 3.3.3. Aging Worsens Diabetic Complications

Due to various factors, such as improvement in medical standards, the global population with diabetes complications shows the characteristics of aging [11]. Aging may have two influences on diabetes complications. On the one hand, there is a higher prevalence of diabetes with age. On the other hand, the prolonged course of diabetes in aging patients often leads to a greater possibility of vascular complications. The multicountry results of diabetes complications compiled by Beckman showed that the prevalence of various diseases, including the peripheral vascular complications PAD and DM retinopathy, increases with aging [53]. A study of risk factors in China also found that increasing age greatly increases the incidence of PAD: 2.81% (95% CI = 1.77 − 4.43) and 3.84% (95% CI = 2.44 − 5.98), respectively, in men and women aged 25-29 compared with 21.95% (95% CI = 15.39 − 30.31) and 27.95% (95% CI = 20.14 − 37.37) in men and women aged 95-99 years [54].

#### 3.3.4. Gestational Diabetes

In developing regions, the follow-up impact of gestational diabetes (GDM) has gradually received attention because the disease may not only affect the pregnant women themselves but also have a profound impact on fetal diabetes metabolism and disease [55]. Indian research has pointed out that the prevalence of type 2 diabetes for mothers with GDM is increased 7-fold and that the fetus will be more likely to have obesity or T2DM [17]. A pooled study in China showed that in a group of 7-year-old children, the offspring of mothers with GDM were twice as likely to be obese [16]. Because this disease needs to be prevented between generations, the number of affected groups is large, the age of onset is often earlier, and the cost of treatment can be double; therefore, special attention should be paid.

**Figure 1:**
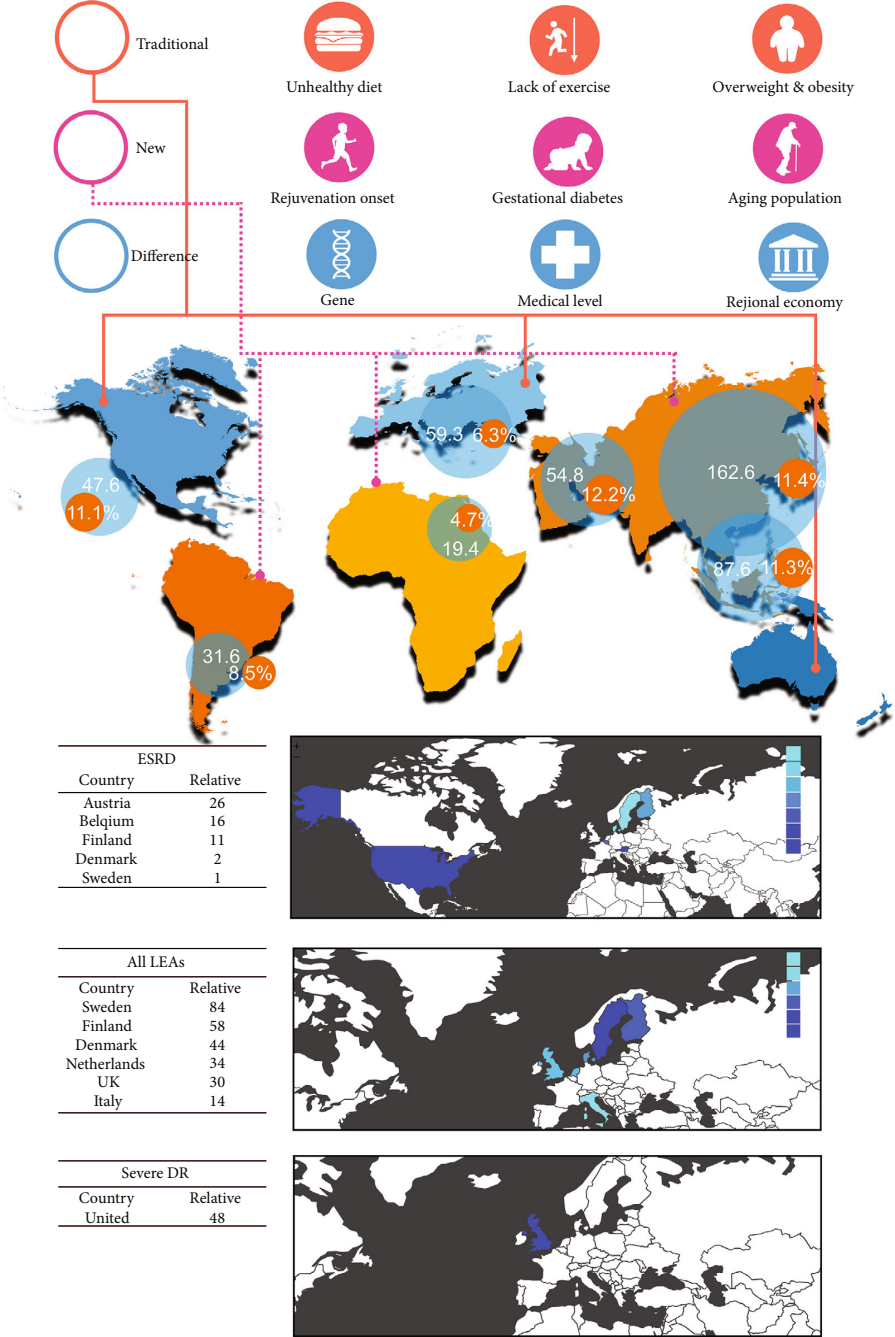
The risk factors for diabetic vascular complications and the decrease in morbidity caused by treatment. Worldwide, the incidence of diabetes has shown an overall upward trend. It is worth noting that there are obvious differences in the incidence of diabetes between different regions, which are presumably caused by differences in economic development, medical care levels, and genetics between regions. Due to an unhealthy diet and lack of exercise, which leads to obesity, current diabetes prevention also needs to pay attention to the effects of dietary changes in some areas, childhood obesity, gestational diabetes, and population aging. Fortunately, better medical treatment can bring about improvements in the incidence of diabetic vascular complications. (For the convenience of observation, the connection between Europe and Asia is split. The data in the picture come from an article that analyzes the changes in IDF and vascular complications [1, 11].)

Figure 1 shows a comparison of risk factors for diabetes in different regions, the incidence of diabetes, and the decline in diabetic vascular complications in Europe (Figure 1).

## 4. Macrovascular Complications

Diabetic macrovascular disease is the main way that diabetes reduces the life of diabetic patients. Macrovascular disease of important organs is likely to cause disability and even death in patients. The lesions of macrovascular disease can be mainly divided into cardiovascular, cerebrovascular, and peripheral arterial lesions [8]. A pooled study of 57 reports worldwide found an overall macrovascular complication rate of 32.2% among 4,549,481 patients with T2DM who were systematically reviewed [56].

A comparison of the risk for different diabetic vascular complications is shown in Table 1.

### 4.1. Cerebrovascular Disease

An important manifestation of diabetic cerebrovascular disease is stroke. In addition to vascular disease in the brain, it may also be related to insufficient blood supply to the brain caused by carotid artery disease. The INTERSTROKE study, which included stroke-related data from 32 countries, showed that compared with the nondiabetic population, the risk of stroke in diabetic patients was increased by 16% [57]. Guo et al. also found that among diabetic patients in Zhejiang Province, China, from 2007 to 2013, the risk of stroke in both male and female type 2 diabetic patients was more than three times greater than that in the nondiabetic population. Among them, women (OR = 3.87, 95%; CI = 3.76 − 3.99) had an obviously higher risk than men (OR = 3.38, 95%; CI = 3.27 − 3.48) [58].

Compared with normal patients, diabetic patients have poorer outcomes from stroke because diabetes mediates both vascular disease and neuropathy, exacerbating neurological sequelae, such as recurrence and death [59]. A follow-up study conducted in China showed that among the 143 people surveyed, patients with uncomplicated diabetes were more likely to have stroke recurrence than nondiabetic individuals. The risk was increased by 2.77 times (HR = 2.77, 95% CI = 1.66 − 4.63) [60]. A study combining the Emerging Risk Factors Collaboration (ERFC) and the British Biobank showed that compared with stroke alone (mortality per 1,000 person-years = 15.6), diabetes significantly increased the mortality rate of stroke (diabetes and stroke deaths per 1,000 person-years rate = 32.5) [61]. Among them, the course of disease may be an important factor affecting the prognosis. A study analyzing German medical insurance pointed out that between 2005 and 2009, the malignant degree of stroke increased in the 5,757 patients under investigation due to the prolonged course of diabetes. Ultimately, 1/3 of the patients died of cerebrovascular disease within 5 years [62].

Fortunately, the control of cerebrovascular disease in developed areas may have a relatively good effect on the incidence of stroke. Many countries have shown a certain downward trend, but similar conclusions may not be applicable in India and China [11, 16, 17].

### 4.2. Cardiovascular Disease

Diabetes patients are prone to secondary heart-related vascular complications. The main manifestations are CHD, myocardial infarction (MI), and sudden cardiac death (SCD). A systematic study by Einarson and colleagues that aggregated 57 data points from all over the world pointed out that 32.2% (53 studies, *n* = 4,289,140) of more than 450,000 patients with type 2 diabetes had diabetic macrovascular disease, of which CVD accounted for 21.2%, followed by heart failure (14.9%), angina pectoris (14.6%), and MI (10.0%), and the probability of occurrence of these cardiovascular diseases was much larger than that of cerebrovascular lesions [56].

In cardiovascular disease, one of the more serious consequences is heart failure. In earlier studies, the impact of diabetes was thought to be similar to that of MI. A Finnish-based population study conducted by Haffner et al. showed that patients with diabetes but no previous MI had the same risk of MI in the future as patients with MI without diabetes; thus, a history of type 2 diabetes can be considered to be equivalent to a previous MI [63]. However, the Finnish study at the time adopted the 1980 diagnostic criteria, which includes a higher blood glucose threshold than the new standard; thus, the degree of diabetes was more serious, which may have exaggerated the effect of general diabetes on cardiovascular disease. A study conducted by Schramm and collaborators in Denmark based on 3.3 million people found that diabetes can indeed increase the risk of neovascular disease (males OR = 2.42, 95% CI = 2.35 − 2.49; females OR = 2.45, 95% CI = 2.38 − 2.51), but this effect was weak in patients with coronary heart disease (males OR = 2.44, 95% CI = 2.35 − 2.49; females OR = 2.62, 95% CI = 2.38 − 2.51) [64]. However, there are also experiments showing that the level of blood glucose control seems to be independent of severe cardiovascular results. A cohort study of 2,740 patients in the United States conducted by de Simone et al. showed that HbA1c levels were higher in patients without heart failure than in those with heart failure (7.92 ± 2.47% vs. 6.78 ± 2.39%, *p* < 0.04); the products of abnormal metabolism in diabetic patients are suspected to be able to directly provide energy for myocardial contractions [64].

According to Beckman, in addition to increasing the risk of cardiovascular disease, diabetes also worsens its consequences [65]. An investigation into frequently occurring cardiometabolic diseases indicated that diabetes may lead to myocardial apoptosis through cell oxidation or endoplasmic reticulum stress. As a result, among patients with diabetes, those with MI (32.0 per 1,000 person-years) are more likely to experience cardiovascular death than those with MI alone (16.8 per 1,000 person-years) [61, 66]. A plasma phospholipid transfer protein knockout (PLTPO) experiment further pointed out that in patients who have undergone PCI treatment, the severity of diabetes may be positively correlated with cardiovascular death because the risk of cardiovascular death was 50% higher in patients with diabetes who received insulin treatment than in those who did not; moreover, the prognosis is worse after normal blood flow is restored by stent placement [67].

Due to genetic differences, cardiovascular disease in Asian populations is different from that in Caucasian populations. A pooled study from India pointed out that from the perspective of the time of onset of the disease, the course of CAD occurs earlier in some Asian populations than in Caucasian populations, and the mortality rate caused by acute macrovascular events is also higher. The main reason may be that Asian people have higher levels of insulin and blood lipids after the onset of type 2 diabetes than Caucasian people, which likely causes more serious insulin resistance and endothelial disorders, further accelerating the course of vascular disease and causing more serious disease [17]. However, on a global scale, due to improvements in medical care in most developed countries, the risk of diabetic cardiovascular disease has gradually decreased. This may be due, in part, to improved blood sugar control in diabetic patients, but it may also be related to a decline in the incidence of CVD itself [11].

### 4.3. Peripheral Vascular Disease and Diabetic Foot

In addition to cerebrovascular and cardiovascular diseases, another important effect of diabetic macrovascular disease is peripheral vascular disease. The main cause of the pathological changes is atherosclerosis of the large blood vessels of the lower extremities, leading to blood circulation disorders. One of the characteristic secondary pathological changes is diabetic foot. A study by Song et al. compiled 37 PAD-related parameters in China and showed that diabetes is the third leading cause of PAD after smoking and hypertension. Patients with diabetes are nearly 1.71 times (95% CI = 1.45 − 2.01) more likely to develop PAD than nondiabetic patients [54].

Diabetes also increases the consequences of PAD because it involves more distant acral vessels and may cause medial sclerosis and accelerate vascular hardening through mechanisms such as oxidative stress, genetic damage, and vascular damage [68]. In addition to vascular disease, diabetes itself can also cause lower limb neuropathy, microvascular disease, and changes in foot dynamics. These factors work together with PAD to cause diabetic foot [69]. For patients, diabetes can worsen the quality of life after PAD onset. Studies have pointed out that compared with the general population, diabetic patients tend to be more prone to lower extremity pain, hypoesthesia, and other sensory changes. This is because the correlation between diabetic vascular disease and neuropathy is approximately 2-fold higher [70].

At present, the control of diabetic peripheral vascular disease remains mixed. A study summarizing lower limb amputation in many European countries showed that the current control situation is still mixed. In Ireland and Spain, among other places, the rates of major amputation among diabetic patients have increased from 0.0479% and 0.00712% to 0.0480% and 0.00747%, respectively, in different time periods; the rates of minor amputations in the corresponding regions increased from 96.2/100,000 and 9.23/100,000 to 127/100,000 and 10.970/100,000, respectively. In addition, the rate of minor amputations in Finland also increased from 11.0/100,000 to 13.5/100,000 [71]. However, a study conducted by Kurowski and others in Western Australia showed that from 2000 to 2010, among a total of 5,981 amputations, as many as 71% of the patients had peripheral arterial disease. Under the premise that the lower extremity amputation rate has dropped overall, diabetic patients (6.2% per year) have shown a smaller decline in the amputation rate than nondiabetic individuals (6.7% per year) [72].

## 5. Microvascular Complications

In addition to macrovascular lesions, microvascular lesions also play an important role in mediating later complications, but microvascular lesions are often easily overlooked. From a diabetes nursing perspective, the degree of microvascular disease is commonly used today to reflect the degree of care.

### 5.1. Kidney Disease

Diabetes is the main cause of diabetic nephropathy, and the end-stage renal disease (ESRD) population is dominated by diabetic patients [73]. Similar to those of retinopathy, the histological manifestations of diabetic nephropathy include thickening of the basement membrane and aneurysms. Along with an increase in glomerular filtration, expansion of the adjacent cell matrix and progression of partial sclerosis of the nephron, the original filtration barrier is destroyed [53].

Zhang et al. compiled reports from 30 studies conducted in China and showed that among the 79,364 participants surveyed, up to 21.8% of diabetic patients (95% CI = 18.5 − 25.4%) had diabetic nephropathy and that the prevalence rate in the region (41.3%) was significantly higher than that in Western countries (22.3%) [74]. In some areas, as many as 7% of initially diagnosed diabetic patients have ESRD, and a survey showed that the prevalence of kidney disease in type 2 diabetic patients reached 25% after 10 follow-up visits [75, 76]. A study conducted by Yeung in Hong Kong showed that among hospitalized patients, the proportions of type 2 diabetic patients with microalbuminuria and macroalbuminuria were 24.9% (95% CI = 22.9 − 27.0%) and 18.3% (95% CI = 16.5 − 20.2%), respectively, showing that nearly 60% of patients with type 2 diabetes may have some degree of kidney disease. In contrast, the prevalence of kidney disease in patients with type 1 diabetes is less than 12% at 7 years after diagnosis [77].

Similar to other vascular diseases, ESRD shows a downward trend in developed areas but has a worsening trend in economically underdeveloped areas. A study of several databases in the United States showed that over 20 years (1990-2010), the incidence of ESRD in diabetic patients decreased by 28% and that the incidence of ESRD in all age groups decreased significantly after 2000 [2]. During the same period, the incidence of diabetes-related ESRD in some Western European countries and Nordic countries declined to varying degrees. In contrast, however, diabetic nephropathy in parts of Russia, East Asia, and South Asia increased rather than decreased [49]. A study by Huang et al. obtained data from 878 hospitals in China and showed that diabetic nephropathy among chronic kidney disease (CKD) patients increased from 19.5% in 2010 to 24.3% in 2015, with an average annual growth rate of 0.96% during the period; this trend was most significant in the populations living in cities in the northern region [78].

### 5.2. Retinopathy

Diabetic patients affected by retinopathy account for 30% of the total retinopathy population, and some patients with retinopathy have a high probability of eventually becoming blind. In different follow-up studies, the incidence of diabetic retinopathy has developed in different directions in people worldwide [11]. At present, retinopathy is usually divided into proliferative and nonproliferative. The main difference between the two is whether there is retinal vascular renewal. However, both types of retinopathy are accompanied by the destruction of the pericytes that wrap the retina, which results in the blood vessels of the retina losing the ability to maintain normal vascular tension, growing unstable, and becoming easily damaged by oxides, ultimately leading to the destruction of the retinal barrier to cause microangiopathy [11]. Song et al. conducted a pooled study in China and showed that the incidence rates of proliferative diabetic retinopathy (PDR) and nonproliferative diabetic retinopathy (NPDR) reached 0.99% (95% CI = 0.40 − 1.80) and 15.06% (95% CI = 11.59 − 18.88) and that the aggregated changes in any type of diabetic retinopathy reached 18.45% (95% CI = 14.77 − 22.43) [79].

From an optimistic point of view, studies from 2000 to 2017 show that the incidence of diabetic retinopathy (STDR) in many regions has dropped significantly, with the highest decline reaching an astonishing 91% (1.7% to 0.16%). In some countries, such as the United Kingdom, the incidence of several different types of retinopathy increased by more than twofold (2.0% to 4.7%) from 1991 to 2006. These declining trends may largely be attributed to better treatment levels and the use of better care measures [80]. However, European studies on future predictions show that the incidence of diabetes in Europe is expected to increase by 2 million people over the next 30 years; approximately 50% of patients with type 1 diabetes and approximately 25% of patients with type 2 diabetes will continue to suffer from diabetic eye disease [81].

### 5.3. Neuropathy

Diabetic neuropathy is related to the mechanism of microvascular disease. Due to the hardening and thickening of the basement membrane of the capillaries, the blood flow of the capillaries that nourish the nerves is reduced, and along with damage to the surrounding cells, leads to continuous hypoxia, oxidative stress, and other complexities. The downstream mechanisms jointly destroy the nervous tissue [82, 83]. Specific types of diabetic neuropathy can be divided into two categories. The first category, which is more common, is called typical diabetic peripheral neuropathy (DPN), also known as length-dependent sensorimotor polyneuropathy (DSPN); the second category is atypical DPN, also called polyneuropathy [84]. The risk factors caused by high blood sugar levels may be more clinically significant in typical DPN, while atypical DPN is rarer and has a less predictable disease course, so it is more difficult to make inferences from risk factors [53]. A study by Lu and colleagues in Shanghai showed that among 2,035 individuals with abnormal blood glucose regulation, including diabetes, impaired glucose regulation (IGR) and normal glucose tolerance (NGT), the incidence of peripheral neuropathy (PN) in patients with known diabetes reached 13.1%, and the incidence in the IGR population was also increased by approximately 1.8 times compared with that of the normal population (2.8% vs. 1.5%) [85]. However, few studies on diabetic vascular complications alone exist because neuropathy is often considered to be an auxiliary cause of diabetes and is analyzed together with microvascular disease.

## 6. Countermeasures for Diabetic Vascular Complications

**Figure 2:**
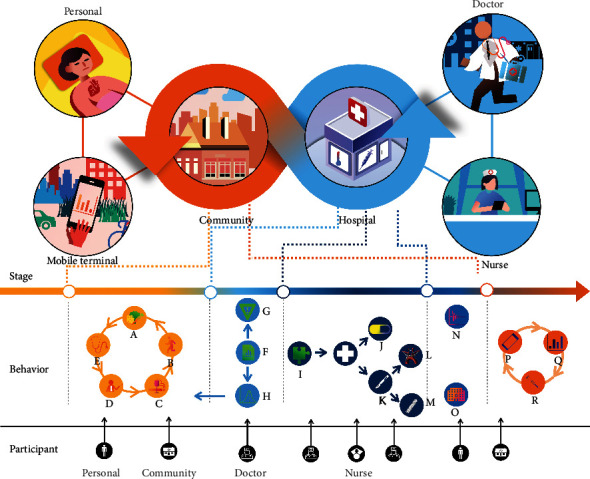
The prevention, treatment, and management of diabetic vascular complications. A: Diet; B: sport; C: quitting smoking and alcohol; D: health knowledge information; E: regular health examinations; F: diagnostic results; G: report individual risk factors; H: aggregate data analyses; I: treatment plans; J: drugs; K: surgery; L: bypass surgery; M: percutaneous coronary intervention; N: index detection; O: hospital care; P: mobile terminals; Q: cloud database analyses; and R: data-based treatment changes. Before the onset of diabetic vascular complications, diabetic patients can reduce their chances of onset by adjusting their diet, increasing exercise, and abstaining from bad habits. At the same time, they can actively participate in physical examinations and health education lectures. Doctors can actively use mobile phone data during the diagnosis and treatment of diabetic vascular complications to obtain accurate risk factors and help prevent them. At the same time, after the treatment is completed, a low-cost and timely chronic disease management system can be established through mobile devices to reduce the risk of recurrence of vascular complications and improve prevention.

In view of the above risk factors and morbidity, we roughly put forward some preventive suggestions at the level of the individual and explored the treatment plan. In response to the needs of patients with diabetic vascular complications, a whole management process from prevention, diagnosis, and treatment to chronic disease care should be established. In this process, patients, communities, and medical staff must participate together (Figure 2).

### 6.1. Lifestyle-Level Prevention

#### 6.1.1. Diet

*(1) Ensure a Reasonable Total Food Intake*. Taking China as an example, the relative intake of the traditional diet should be reduced; this traditional eating pattern can reduce the possibility of obesity [25].

*(2) Maintain a Balanced Nutrient Intake Ratio*. In developed countries, the proportion of coarse grains and vegetables should be increased, thereby increasing the intake of cellulose, maintaining the intake of protein, and reducing the intake of fat. In developing regions, the intake of certain vegetables should be increased to reduce the intake of refined grains, and by consuming affordable meats such as eggs and chicken, the intake of protein should be increased and the intake of fat should be maintained to a certain amount. In some developing countries, the original diet consists of mainly carbohydrates and plants, and therefore, protein accounts for a low proportion of the overall energy intake and grains account for more than 60% of total energy intake [25, 86]. In addition, healthy foods such as vegetables are relatively low priced in China, and thus, it is more feasible to promote a diet that has protective functions against diabetes and cardiovascular diseases [87].

*(3) Reduce the Intake of Sugar-Sweetened Beverages*. Malik and others have pointed out that it is possible to replace sugary beverages with beverages that do not contain fructose, such as those containing new sugar substitutes and conventional beverages such as water, coffee, and fruit juice. However, a prospective study on beverages found that the consumption of fruit juice and beverages containing sugar substitutes increased by 7% (1% to 14%, *I* (2) = 51%) and 8% (2% to 15%, *I* (2) = 64%), respectively. Although there may be some offset factors, these two beverages are still not recommended as alternatives to sugary beverages [88].

*(4) Moderate Alcohol Intake*. A study by Gupta in Singapore found that occasional and frequent drinkers in the group had a lower likelihood of retinopathy, although the generalizability of this conclusion is debatable, as some scholars have pointed out that long-term alcohol consumption can lead to retinopathy and impair the function of large blood vessels [28].

#### 6.1.2. Physical Activity

*(1) If You Already Have Diabetes, Do Planned Exercise*. A pooled study by Kumar et al. clearly pointed out that planned exercise can be used to treat insulin resistance in type 2 diabetes and can reduce HbA1c levels by 0.27 (-0.82 to 2.08), which is very important for vascular complications; thus, exercise may have a considerable protective effect against the complications of diabetes [89]. Taking into account a decline in physical function among diabetic patients, recommended exercise methods should be reasonable and regular.

*(2) Everyone Should Develop Exercise Habits as Soon as Possible to Improve Their Sports Ability*. A study conducted by the U.S. Veterans' Medical Center among elderly patients showed that exercise capacity is negatively correlated with the mortality of type 2 diabetes patients and that people who do not exercise have relatively poor control over the vascular complications of type 2 diabetes and are more likely to develop vascular complications. Thus, exercise can be used to improve an individual's exercise ability and prevent the vascular complications of diabetes [90].

#### 6.1.3. Smoking and Environmental Pollution

*(1) Quit Smoking as Soon as Possible and Reduce Exposure to Air Pollutants through Reasonable Protective Equipment Such as Masks*. Reducing the amount of smoking or quitting smoking has been proven by many studies to have an important effect on the cardiovascular complications of diabetes. It can also have a certain protective effect against nondiabetic vascular complications. At the same time, the isolation of pollutants and some heavy metal elements in the air by necessary means has also been proven to be related to the prevention of cerebrovascular disease in patients with diabetes (the risk factors for air pollution and lead exposure were found to affect 33.4% of diabetic patients) [32].

### 6.2. Prevention, Diagnosis, and Treatment at the Medical Level

#### 6.2.1. Infer Possible Complications, Choose a Reasonable Inspection Method, and Find Undiagnosed Patients as Soon as Possible

Researchers in China found in the latest national survey that among 170,287 participants, the unknown rate of patients was more than 3/5, and more than 1/3 of the population had abnormal glucose homeostasis, indicating a large number of potentially undiagnosed diabetic patients [91]. Due to the longer actual course of disease, this group may have a higher risk of vascular complications than patients with diagnosed diabetes; therefore, it is necessary to diagnose patients with hidden diabetes in a timely manner.

In terms of macrovascular complications, diabetes also causes certain difficulties in the diagnosis of concomitant blood vessel complications, so it is necessary to choose an appropriate diagnostic method. Approximately 20-30% of patients with type 2 diabetes will experience “silent ischemia,” causing the initial diagnosis of cardiovascular disease to be made after a longer disease duration. Moreover, the disease progression of diabetic patients is also relatively rapid, which worsens the outcome and prognosis of complications [92]. However, some scholars believe that asymptomatic coronary artery disease does not necessarily bring serious consequences based on the screening results of ischemia detection in diabetic patients (DIAD) and CT angiography [93]. In addition, taking PAD as an example, the ankle-brachial index is usually used to measure the degree of peripheral arterial disease. Generally, the critical value of the ankle-brachial index is 0.90. Diabetic patients with an ankle-brachial index less than this value may have a greater risk of peripheral arterial disease. Diagnostic medical ultrasound (CDU) and multidetector computed tomography (CTA) are also commonly used diagnostic methods [94].

Microvascular complications also have many specific effects. Currently, diabetic nephropathy is often diagnosed by proteinuria, but newly discovered specific markers should also be considered. Mannose-binding lectin (MBL) can trigger downstream inflammation and complement pathways, and it has been experimentally proven that after correcting for these factors, MBL is strongly correlated with type 2 (OR = 7.55; 95% CI = 3.44 − 19.04) and type 1 (OR = 6.99; 95% CI = 2.83 − 17.15) diabetes. It is speculated that the MBL level can also be used to predict the possibility of nephropathy [95, 96]. There are also a variety of diagnostic methods for neuropathy, most commonly reflex tests, symptom score tables, and skin puncture. Because neuropathy can also bring pain, convulsions, and other neurological symptoms, these symptoms also need targeted treatment [83].

#### 6.2.2. Control Blood Sugar, Blood Lipids, and Blood Pressure within a Reasonable Range

Manuscripts should make recommendations for glycemic control, blood pressure, and lipid control to prevent cardiovascular complications in diabetic patients. Many scholars have demonstrated that well-controlled blood glucose levels can prevent vascular complications and reduce consequences. A study by Low Wang et al. found that better control of blood glucose levels was associated with less malignant outcomes in peripheral vascular disease, with each percentage point reduction in HbA1c reducing the likelihood of adverse cardiovascular events by 14.2% [97]. Similarly, optimal glycemic control may prevent the development of diabetic nephropathy. A study on multifactor intensive blood glucose treatment showed that after strict blood glucose control and treatment with renal active drugs, the incidence of different kidney diseases in type 2 diabetic patients decreased to various degrees, and the risks of ESRD, microalbuminuria, and macroproteinuria were reduced by 65% (20 events vs. 7 events), 9% (1298 patients vs. 1410 patients), and 30% (162 patients vs. 231 patients), respectively [98]. Lower blood sugar levels are also an important protective factor for retinopathy. Chronic but well-controlled blood sugar levels have been found to reduce the likelihood of retinopathy by 11 times compared to approximately 2 times for coronary artery disease [99]. Additionally, similar to other pathologies, strict blood sugar control and a good lifestyle have also been shown to be effective in preventing neuropathy [100].

Similarly, lowering blood lipids is associated with the prevention of vascular complications, but the specific protective effect of these complications remains controversial. A study conducted in Shanghai, China, showed that diabetic patients with lower blood lipid levels had a lower prevalence of diabetic nephropathy (DKD) but no significant difference in the prevalence of diabetic retinopathy (DR). Of these, TG, TG/HDL-C, and non-HDL-C/HDL-C values were independently associated with diabetes [101].

Numerous studies have also reported that good blood pressure control can reduce the consequences of vascular complications. For example, control of high blood pressure has some benefit in the prognosis of cerebrovascular disease. In a preventive study of cardiovascular complications in more than 3,500 diabetic patients, the incidence of stroke decreased by 33% in patients treated with ACEIs [102].

In summary, although there are certain controversies in clinical trials, the study by Hewitt et al. still recommends controlling blood sugar, blood lipids, and blood pressure as a means to prevent specific complications of diabetes [103].

#### 6.2.3. Appropriate Treatment for Specific Complications

*(1) For the Onset of Cerebrovascular Disease, the First Priority Is to Control Blood Sugar, and at the Same Time, Thrombolytic Drugs Must Be Reasonably Selected According to the Indications*. The prevailing view is that blood supply to the brain can be improved with thrombolytic drugs, but some current research suggests that restoring blood flow to the brain may bring about worse outcomes. A study of thrombolytic drugs showed that among 389 male patients, higher admission blood glucose levels were associated with a poorer prognosis and could cause intracranial hemorrhage. This indicates that ischemia–reperfusion may have a certain destructive effect on cerebrovascular diseases; therefore, the cerebrovascular complications of diabetes should be treated with blood sugar improvements and more conservative strategies [104].

*(2) For Cardiovascular Disease, Drugs Are Recommended for Mild Cases, and Coronary Artery Bypass Graft Therapy Is Recommended for Severe Cases Rather than Interventional Therapy*. Schmidt's review presents the current glucagon-like peptide 1 receptor agonists (GLP-1 RAs), sodium glucose cotransporter-2 (SGLT-2) inhibitors, proprotein convertase subtilisin type 9 (PCKSK9), and other new drugs for the treatment of CVD diseases. In addition, gene-level treatment via RNA-based therapies is awaiting research [105]. In addition, more mainstream CVD treatment can be achieved through vascular stents or bypass surgery. However, due to the presence of diabetes, the measures taken to revascularize may need to be changed. Experiments have proven that the ideal treatment should be vascular bypass surgery rather than implantation of percutaneous stents. Based on research in British Columbia, CABG has a better prognosis than PCI, and the advantage of CABG is more obvious in patients with major adverse cardiac or cerebrovascular events (MACCEs) (0.4995%, 95% CI = 0.34 − 0.71) [106]. Similar findings were found in Ishihara et al.'s study in Japan. After implantation of drug-eluting stents (DES) and dual antiplatelet therapy (DAPT), diabetic patients had more uncovered struts in the short term, and the treatment was not ideal [107]. A study conducted by Ramanathan from 1982 to 2011 proved that cardiac bypass has a considerable effect on survival. At 1, 5, 10, and 20 years, the survival rates could reach 97%, 97%, 96%, and 96%, respectively, all exceeding 95% [108].

*(3) For Peripheral Vascular Disease, Especially Lower Extremity Vascular Disease, Drug Therapy Is Recommended, Followed by Vascular Surgery, and New Treatments Such as Endovascular Lithotripsy Should Be Selected According to the Situation*. Similar to the treatment of cardiovascular diseases, there are studies on improving the blood supply of the lower extremities through surgery in the field of diabetes PAD treatment, such as percutaneous stents or vascular bypass. On the premise, PCI is less invasive and has a good prognosis, while the perioperative mortality rate of bypass surgery is as high as 3% [109]. A study by Arvela et al. on the single-segment great saphenous vein (ssGSV) showed that even if bypass angiogenesis of an autologous vein graft (AAVG) is used, there is a higher possibility of vascular stenosis after blood flow is restored (occlusive transplantation failure AAVG vs. ssGSV RR = 2.00, 95%CI = 1.39 − 2.88, *p* < 0.0001) [110]. Taylor et al.'s study in the United States of patients with severe PAD showed that after percutaneous transluminal angioplasty (PTA) in 314 subjects, the loss of walking function and the loss of independence of the lower limbs were improved (HR = 0.53; *p* = 0.025; HR = 0.53; *p* = 0.025); however, the mortality rate was higher than that of conventional amputation (HR = 1.62, *p* = 0.006) [111]. We speculate that because vascular lesions of the lower extremities involve mixed lesions of large and microvessels, interventional surgery may not be ideal for curing microvascular lesions. Therefore, for the treatment of diabetic foot, bypass is not the mainstream treatment. We recommend nonvascular therapy for injured patients, which mainly refers to nursing care, debridement, and taking certain thrombolytic drugs [53]. A study on thrombolytic drugs by Olinic et al. showed that after the use of clopidogrel and aspirin DAPT or aspirin single antiplatelet therapy (SAPT), thrombin receptor antagonists (vorapaxar) can improve the prognosis of PAD, but the risk of bleeding will increase as long as the drug is taken. If patients have undergone surgery, DAPT should also be used for a period of time; patients implanted with transinguinal stents should use DAPT for at least 4 weeks, while those with transknee stents should take DAPT for a longer period [112]. Compared with conventional medical treatment, intravascular lithotripsy (IVL) has also been proven to be more effective. The Disrupt PAD III study conducted in the United States showed that between 2017 and 2018, the prognosis of patients with IVL did not worsen, so this therapy may become a direction for PAD treatment in the future [113].

#### 6.2.4. Good Nursing Care Should Be Provided for Diabetic Patients with Complications

Thankfully, the incidence of CVD events has declined in the developed world as a whole due to better care and revascularization techniques. A survey of vascular complications conducted by Wu in Hong Kong showed that between 2001 and 2016, the incidence of heart failure in diabetic patients decreased by approximately 63.6% (-6.4, 95% CI = −8.0 − −4.7) [114]. Furthermore, a study comparing the clinical experience in the United Kingdom and Sweden also proved that during the period from 2004 to 2010, good treatment had a protective effect against the consequences of cardiovascular complications [115].

### 6.3. Chronic Disease Management

The onset prevention and prevention of diabetes complications is relatively long. Therefore, only treating the initial disease cannot completely control it. Diabetes must be managed after the initial onset, and vascular complications must be promptly targeted as they occur by monitoring risk factors. Among developing countries, India has relatively rich experience in the use of low-cost methods for chronic disease management, such as text messages to provide advice on lifestyle habits for diabetes protection (HR = 0.64, 95% CI = 0.45–0.9, *p* = 0.015) [17]. Similarly, in China, where smart mobile terminals are more popular, medical staff use mobile phone applications to monitor diseases. In a study conducted by Zhang, compared with the control group, the groups using the chronic disease management system had lower levels of glycosylated hemoglobin at the 3rd and 6th months (both *p* < 0.05), and interactive procedures had better results at the 6th month (*p* = 0.04) [116]. In addition, in Europe and the United States, where the field of diabetes management is relatively advanced, digital health technology and related equipment are widely used. The more common methods are continuous blood glucose monitoring systems for testing and insulin pumps or delivery systems that focus on treatment. The popularity of emerging mobile terminals in these regions and the importance of health-related applications are also worthy of attention [117]. The future goals of the management of diabetic vascular complications are the realization of remote diagnosis of disease through computer-related technology, and for the purpose of disease prevention, data sharing can be achieved in hospitals and communities in different urban areas.

## 7. Conclusion and Outlook

This manuscript focused on the macro and microvascular complications of diabetes, and a summary analysis was used to draw some key conclusions: the high prevalence of diabetes in emerging economies and the relative lack of treatment technologies make diabetic patients more prone to vascular complications. Further, the relatively low awareness and compliance among the patients make the prevention of vascular complications even more difficult. A sample survey found that there is a large number of undiagnosed diabetic patients in the population. Although the prevalence of diabetic vascular complications in developed countries and regions has shown a trend of relative improvement, the resources consumed by the corresponding better medical technology will place a heavy burden on society. There are also many new challenges in the prevention of diabetic vascular complications. For example, a younger generation of diabetic patients has increased the incidences of related diseases. At the same time, the aging of the global population makes it difficult to reduce the prevalence of diabetes, and many studies have reported an expansion in the impact of gestational diabetes. To prevent and control the occurrence and development of diabetes complications, multistep cooperation from medical workers to patients is needed. At the same time, actions should be taken by the government and other relevant departments for disease prevention. The onset of diabetes itself and its vascular complications can be additionally controlled through the following measures: (1) In terms of lifestyle factors, diabetic patients are encouraged to reduce their total caloric intake, decrease the proportions of oil and salt and increase the proportions of fiber-rich foods and high-quality protein in their diet, participate in appropriate physical exercise, and reduce the frequency of smoking and alcohol consumption. (2) In terms of treatment, patients with milder illness should strictly control their blood sugar, blood lipids, and blood pressure. Those with severe vascular complications should actively receive drug treatment, necessary surgical treatment, and better diabetes care practices. (3) Clinicians should adopt a chronic disease management system for timely follow-up and monitoring of disease and provide personalized control plans based on the principles of precision medicine.

## Figures and Tables

**Table 1:** Comparison of the risk of different diabetic vascular complications.

Event	Country/region	Research time period	Number of overall research participants	Specific event	Evaluation parameters	95% CI
Cerebrovascular disease	China [70]	2003~2004	1,087	Stroke recurrence	HR = 2.77	1.66-4.63
	Worldwide [63]	2007~2015.8.8	40,391	All stroke	OR = 1.16	1.05-1.30
	Worldwide [67]	1966~2013	787,924	Diabetes-related stroke	Females RR = 2.28; males RR = 1.83	1·93-2·69; 1·60-2·08
	China [64]	2007~2013	635,252	Stroke and stroke subtypes	Females SIR = 3.87; males SIR = 3.38	3.76-3.99; 3.27-3.48
	Iran [65]	1976~2002	116,316	All stroke	T1DM females RR = 4.7; T2DM females RR = 1.8	3.3-6.6; 1.7-2.0
	Japan [66]	1990~2004	35,747	Ischemic stroke	HR = 4.64	1.76-12.2
	Germany [69]	2005~ 2007	5,757	Risk of death after stroke (30 days, 1-2 years, 3-5 years)	Mortality rate: HR = 0.67; HR = 1.42; HR = 1.00	0.53-0.84; 1.09-1.85; 0.67-1.41
Cerebrovascular disease	China [72]	2007~ 2008	22,216	Death 6 months after ischemic stroke	OR = 1.23	1.10-1.37
Cardiovascular disease	Worldwide [73]	1966~2013	886,710	Coronary heart disease	Females RR = 2.82; males RR = 2.16; females vs. males RRR = 1.44	2.35-3.38; 1.82-2.56; 1.27-1.63
	China [74]	1986~ 2009	110,660	Cardiovascular death	Females HR = 6.9; males HR = 3.5	—
	Finland [77]	1982~ 1984	—	Myocardial infarction	HR of death due to coronary heart disease among non-DM patients with a previous MI history vs. DM patients without a previous MI history = 1.0	0.7-2.6
	Denmark [79]	1997-2002	—	Cardiovascular death	HR of DM males without a previous MI history = 2.42; HR of non-DM males with a previous MI history = 2.44	2.35-2.49; 2.39-2.49
	Sweden [83]	—	18,624	Primary composite endpoint; all-cause mortality; stent thrombosis; and major bleeding	DM patients: HR = 0.88; HR = 0.82; HR = 0.65; HR = 0.95	0.76-1.03; 0.66-1.01; 0.36-1.17; 0.81-1.12
Peripheral vascular disease	China [85]	1990-2000	—	PAD	OR = 1.71	1.45-2.01
	Worldwide [86]		13,885	PAD	ARD = 5.5%; OR = 1.43	1.28-1.61

## Data Availability

All relevant data are available in this paper.

## Additional Points

*New and Noteworthy*. This manuscript focused on the macro and microvascular complications of diabetes, and a summary analysis was used to draw some key conclusions: the high prevalence of diabetes in emerging economies and the relative lack of treatment technologies make diabetic patients more prone to vascular complications, and the relatively low awareness and compliance among the patients make the prevention of vascular complications even more difficult. The resources consumed by the corresponding better medical technology will place a heavy burden on society in developed countries. There are also many new challenges, such as gestational diabetes, juvenile onset, and aging that worsen diabetic complications. To prevent and control the occurrence and development of diabetes complications, multistep cooperation from medical workers to patients is needed.
